# Telomere Length in Adolescent and Young Adult Survivors of Childhood Cancer

**DOI:** 10.3390/cancers16132344

**Published:** 2024-06-26

**Authors:** Meerim Park, Dong-Eun Lee, Yuna Hong, Jin Kyung Suh, Jun Ah Lee, Myungshin Kim, Hyeon Jin Park

**Affiliations:** 1 Department of Pediatrics, Center for Pediatric Cancer, National Cancer Center, Goyang 10408, Republic of Korea; meerim@ncc.re.kr (M.P.); jksuh@ncc.re.kr (J.K.S.);; 2 Biostatic Collaboration Team, Research Institute, National Cancer Center, Goyang 10408, Republic of Korea; 3 Catholic Genetic Laboratory Center, Seoul St. Mary’s Hospital, College of Medicine, The Catholic University of Korea, Seoul 06591, Republic of Korea; yuna1013@catholic.ac.kr; 4 Department of Laboratory Medicine, College of Medicine, The Catholic University of Korea, Seoul 06591, Republic of Korea

**Keywords:** telomere, adolescent and young adult, childhood cancer, survivor

## Abstract

**Simple Summary:**

Leukocyte relative telomere length (RTL) has not been thoroughly studied among childhood cancer survivors, although survivors are believed to be at risk of telomere attrition due to exposure to cytotoxic cancer treatments. We examined the leukocyte relative telomere length (RTL) in 88 adolescent and young adult childhood cancer survivors. Overall, RTL was not significantly shorter than in age-matched references. However, among 43 patients with hematologic malignancies, those who underwent allogeneic hematopoietic stem cell transplantation had significantly shorter RTL, especially if they developed acute graft-versus-host disease ≥ grade II. Patients with severe or multiple chronic health conditions also had shorter RTL.

**Abstract:**

We examined the leukocyte relative telomere length (RTL) in Korean adolescent and young adult (AYA) survivors of childhood cancer and evaluated the association of leukocyte RTL with multiple factors, including malignancy type, cancer treatment, age, and chronic health conditions (CHCs). Eighty-eight AYA survivors of childhood cancer with a median follow-up period of 73 months were recruited. RTL in pediatric cancer survivors was not significantly shorter than the predicted value for age-matched references. Neither age at diagnosis nor duration of therapy influenced the RTL. Among the 43 patients with hematologic malignancies, those who underwent allogeneic hematopoietic stem cell transplantation (HSCT) showed a significant shortening of the RTL compared with those who did not (*p* = 0.039). Among the 15 patients who underwent allogeneic HSCT, those who developed acute graft-versus-host disease (GVHD) of grade II or higher had significantly shorter RTL than those who did not (*p* = 0.012). Patients with grade II CHCs had significantly shorter RTL than those without CHCs or with grade I CHCs (*p* = 0.001). Survivors with ≥2 CHCs also exhibited shorter RTL (*p* = 0.027). Overall, pediatric cancer survivors had similar telomere lengths compared to age-matched references. HSCT recipients and patients with severe or multiple CHCs had shorter telomeres. GVHD augmented telomere attrition in HSCT recipients.

## 1. Introduction

Telomeres are sequences of non-coding repeated deoxyribonucleic acid (DNA) located at the ends of chromosomes. They play a critical role in maintaining chromosome stability and integrity. The length of leukocyte telomeres in humans, which is usually a few thousand base pairs, decreases by 22–45 base pairs per year and is inversely correlated with a person’s chronological age [1]. The telomere length in hematopoietic cells reflects the overall maintenance of telomeres in other tissues and is a reliable indicator of aging at both the cellular and organism level [2]. The shortening of telomeres has been reported following chemotherapy and/or hematopoietic stem cell transplantation (HSCT) [3]. Moreover, telomere shortening has been correlated with chemotherapy intensity [4,5]. However, the literature shows mixed results regarding the correlation between cancer treatment and telomere length changes. While some studies indicate a decrease in telomere length following treatment, others have found no such association [6,7].

Childhood cancer survivors may be at a higher risk for telomere attrition due to the lasting biological damage caused by chemotherapy. Additionally, frailty in this population is linked to a higher risk of developing late effects, indicating that childhood cancer survivors may experience accelerated aging [8]. Leukocyte relative telomere length (RTL) has not been thoroughly studied among childhood cancer survivors, although survivors are believed to be at risk of telomere attrition due to exposure to cytotoxic cancer treatments. The impact of therapy-related telomere shortening on clinical outcomes has yet to be determined. As a significant number of children who have been successfully treated for cancer in the past thirty years are predicted to live a normal lifespan, the evaluation of telomere length in long-term survivors of childhood cancer is of particular importance.

In this study, we aimed to examine the leukocyte relative telomere length (RTL) of adolescent and young adult (AYA) survivors of childhood cancer and evaluate their association with multiple factors, including malignancy type, cancer treatment, age, and chronic health conditions (CHCs).

## 2. Materials and Methods

### 2.1. Study Population

This study was conducted at a single center in a retrospective cohort of survivors of childhood cancer diagnosed between 2000 and 2021 with a longitudinal follow-up of ≥1 year after the cessation of treatment. The peripheral blood (PB) samples were collected between June 2022 and January 2023. This study enrolled 88 survivors of childhood cancers with the following criteria: diagnosis before the age of 19 years, continuous maintenance of clinical remission, compliance with regular clinical follow-up, and cessation of treatment more than one year before the start of the study. Considering that telomeres shorten with age, this study limited participants to those between 15 and 35 years of age at blood sampling. We confined the study participants to Koreans to enhance ethnic and racial homogeneity. Individuals who were pregnant or had received any vaccinations within the last six months were not included. All participants provided informed consent, and the study was approved by the Institutional Review Board of the National Cancer Center (NCC2022-0195).

### 2.2. Clinical Data

Cancer treatments included chemotherapy, radiotherapy, and HSCT. Trained research staff extracted the data on cancer treatment and CHCs from the medical records. CHCs were defined using the modified Common Terminology Criteria for Adverse Events (version 4.0). The CHCs were graded as 0 (no problem), 1 (mild), 2 (moderate), 3 (severe/disabling), 4 (life-threatening), or 5 (death).

### 2.3. Measurement of Telomere Length via Flow FISH

In clinical settings, telomere fluorescence in situ hybridization and flow cytometry (flow FISH) are more accurate, reproducible, sensitive, and specific than quantitative polymerase chain reactions (qPCR) in the measurement of human leukocyte telomere length [9]. The telomere PNA Kit/Fluorescein isothiocyanate (FITC) for flow cytometry (Agilent Technologies, Singapore) was utilized for flow FISH. The method described by Behrens et al. [10] was employed in this study. The control cell line 1301 was used as a reference for calculating the RTLs. Flow FISH involves staining with FISH probes and antibodies for flow cytometry measurements. The protocol was followed as per the manufacturer’s instructions (Telomere PNA Kit/FITC Code K5327, Revision 2020.11). For telomere length measurement with respect to DNA content, preparations including unstained controls were required per patient, consisting of 2 × 10^6^ of both sample and control cells. Fluorescence was measured using a FACSLyric instrument (BD Biosciences, Franklin Lakes, NJ, USA). RTL was calculated by comparing the mean fluorescence intensities of the sample cells to control cells, with blank values subtracted, following the manufacturer’s instructions (Telomere PNA Kit/FITC for Flow Cytometry, Agilent Technologies Singapore).

$$\mathrm{RTL}=\frac{\left(\mathrm{mean}\;\mathrm{FL}1\;\mathrm{sample}\;\mathrm{cells}\;\mathrm{with}\;\mathrm{probe}-\mathrm{mean}\;\mathrm{FL}1\;\mathrm{sample}\;\mathrm{cells}\;\mathrm{without}\;\mathrm{probe}\right)\times \mathrm{DNA}\;\mathrm{index}\;\mathrm{of}\;\mathrm{control}\;\mathrm{cells}\times 100}{\left(\mathrm{mean}\;\mathrm{FL}1\;\mathrm{control}\;\mathrm{cells}\;\mathrm{with}\;\mathrm{probe}-\mathrm{mean}\;\mathrm{FL}1\;\mathrm{control}\;\mathrm{cells}\;\mathrm{without}\;\mathrm{probe}\right)\times \mathrm{DNA}\;\mathrm{index}\;\mathrm{of}\;\mathrm{sample}\;\mathrm{cells}}$$

### 2.4. Statistical Analysis

The patient demographic data and characteristics were expressed as frequencies with percentages or medians with minimum–maximum ranges. Linear regression was performed, and the correlation coefficients were calculated to determine the relationship between age at the time of blood collection and telomere length. Individual variances were assessed by comparing the RTLs predicted using the regression line and the observed values in a residual plot. The telomere length was compared according to the clinical factors using the Wilcoxon rank-sum test or the Kruskal–Wallis test. A two-way analysis of variance (ANOVA) was performed on the rank-transformed data to examine the differences among the telomeres according to the patients’ diagnosis and age. Statistical significance was set at a *p* value of <0.05, and statistical analyses were performed using the R project for statistical computing (version 4.1.2).

## 3. Results

### 3.1. Characteristics of the Study Population

A total of 88 AYA survivors of childhood cancer were recruited during the study period. The clinical characteristics of the patients are shown in Table 1. The median age at the time of blood sampling was 22.5 years (range, 15–34). Survivors were diagnosed with cancer at a median age of 13.0 years (range, 1–19) and were recruited at a median of 73 months (range, 12–184) after the cessation of treatment. Survivors were 53.4% male and had leukemia/myelodysplastic syndrome (*n* = 31, 35.3%), lymphoma (*n* = 12, 13.6%), a brain tumor (*n* = 16, 18.2%), sarcoma (*n* = 14, 15.95%), a germ cell tumor (*n* = 12, 13.6%), or other malignancies (*n* = 3, 3.4%). Among 43 survivors of hematological malignancies, 15 (34.8%) underwent allogeneic HSCT. Of them, acute and chronic graft-versus-host disease (GVHD) were observed in seven (46.6%) and six patients (40.0%), respectively. Donors for patients who underwent allogeneic HSCT had a median age of 23 years (range, 10–39) and did not significantly differ from the age of patients upon study enrollment (*p* = 0.803).

Relapse occurred in six patients with hematologic malignancies and eight patients with solid tumors, while four of the six patients with relapsed hematologic malignancies underwent allogeneic HSCT.

### 3.2. Telomere Length in Survivors

**Figure 1:**
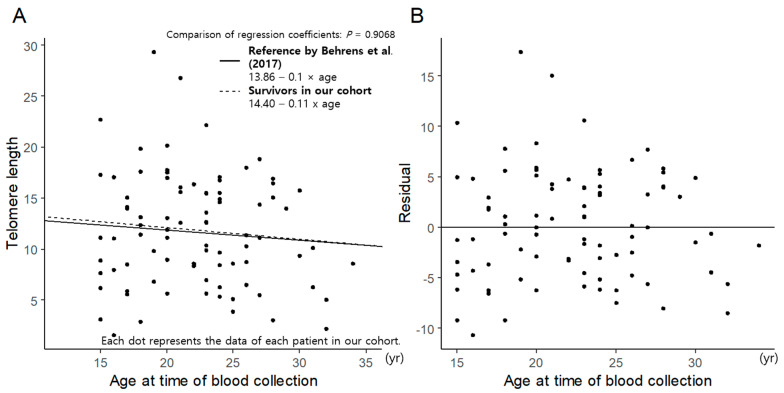
Telomere length according to age: (**A**) comparison between the study cohort and age-matched reference (Behrens et al. [10]) and (**B**) residual plot.

The telomere length measured via flow FISH according to age is shown in Figure 1A. To assess the differences in telomere length between childhood cancer survivors and age-matched references, we compared the linear regression plot of telomere length in survivors in this study with that in an age-matched reference group from a study by Behrens et al. [10] (Figure 1A). There was little difference between the linear regression plots of the two groups. To assess for individual variance, we plotted the residual, which is the difference between the observed and estimated telomere length, for all survivors. The residuals were randomly distributed above and below the zero line, and the distribution was extremely wide, at −10 to 15, indicating that the observed telomere variance was relatively large (Figure 1B).

**Figure 2:**
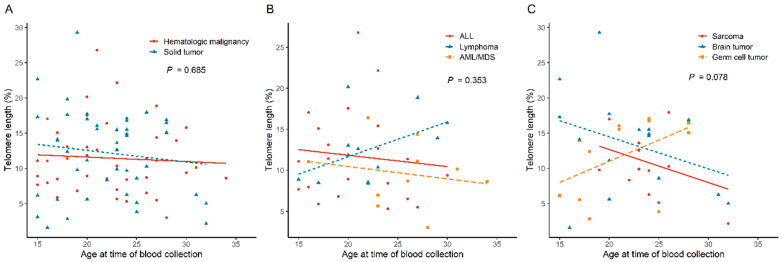
Association between age at blood collection and telomere length: (**A**) comparison between patients with hematologic malignancies and those with solid tumors, (**B**) subgroup analysis of patients with hematologic malignancies, and (**C**) subgroup analysis of patients with solid tumors. ALL, acute lymphoblastic leukemia; AML, acute myeloid leukemia; MDS, myelodysplastic syndrome.

No significant difference was observed in the telomere length between patients with hematologic malignancies and those with solid tumors (*p* = 0.685). In a subgroup analysis of patients with solid tumors, the RTL decreased with age in patients with sarcomas and brain tumors but not in those with GCTs, but the *p* value was not significant (*p* = 0.078) (Figure 2).

**Figure 3:**
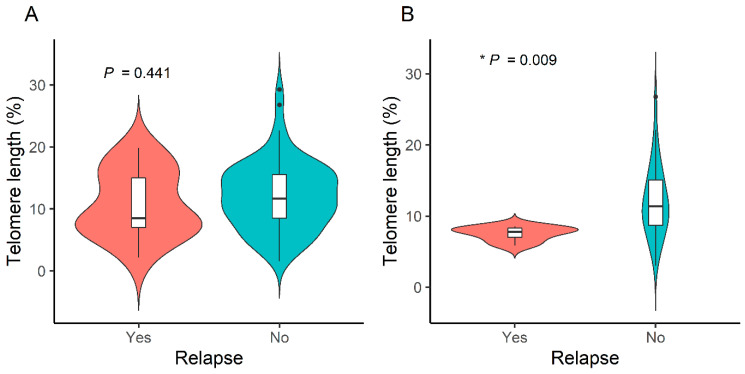
Telomere length according to relapse status in (**A**) all patients and (**B**) patients with hematologic malignancies, * *p* < 0.05.

Overall, relapse showed no association with RTL (10.8% ± 5.4% with relapse vs. 12.0% ± 5.5% without relapse, *p* = 0.441) (Figure 3A). However, a subgroup analysis was performed in 43 patients with hematologic malignancies; those who experienced relapse had significantly shorter RTL than those who did not (7.6% ± 1.0% vs. 12.1% ± 5.0%, * *p* = 0.009) (Figure 3B). Considering that four of six patients with relapsed hematologic malignancies underwent allogeneic HSCT and an adjusted analysis of transplantation was performed, it was difficult to confirm the statistical significance due to the small sample size.

Neither age at diagnosis nor duration of therapy influenced RTL (*r* = −0.059 and *r* = 0.025, respectively). There was no association between the duration after the end of cancer therapy and RTL (*r* = −0.030).

### 3.3. Telomere Length and Cancer Treatment 

A total of 38 patients (43.2%) received radiotherapy. No significant difference was observed in the RTL between survivors who received radiotherapy and those who did not receive radiotherapy (11.7% ± 5.8% vs. 11.9% ± 5.2%, *p* = 0.869). It was not possible to evaluate the influence of craniospinal irradiation (CSI) on RTL as only 2 of the 16 brain tumor survivors did not receive CSI. 

Six patients underwent high-dose chemotherapy with autologous HSCT, and their RTL did not differ from that of patients who did not (14.3% ± 7.7% vs. 12.2% ± 5.4%, *p* = 0.125). It was not possible to evaluate the impact of anthracyclines or alkylators on the telomere length because >70% of the survivors were exposed to these agents. 

**Figure 4:**
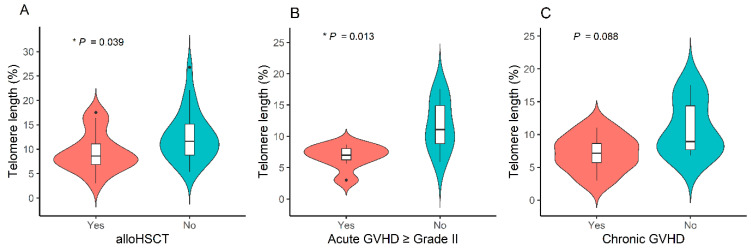
Telomere length according to the presence or absence of (**A**) allogeneic hematopoietic stem cell transplantation, (**B**) acute graft-versus-host disease ≥ grade II, and (**C**) chronic graft-versus-host disease, * *p* < 0.05.

Among 43 patients with hematologic malignancies, those who underwent allogeneic HSCT showed significant shortening of RTL compared with those who did not (9.4% ± 4.1% vs. 12.6% ± 5.1%, * *p* = 0.039) (Figure 4A). Total body irradiation (TBI) during pre-transplant conditioning did not influence the RTL (9.5% ± 3.8% with TBI vs. 12.1% ± 5.1% without TBI, *p* = 0.117). Among 15 patients who underwent allogeneic HSCT, those who experienced acute GVHD grade II or more had significantly shorter RTL than those who did not (6.8% ± 1.9% vs. 11.7% ± 4.1%, * *p* = 0.013) (Figure 4B). Patients with chronic GVHD tended to have shorter RTL than those without, but this difference was not significant (7.1% ± 2.8% vs. 10.9% ± 4.2%, *p* = 0.088) (Figure 4C).

### 3.4. Telomere Length and Chronic Health Conditions

**Figure 5:**
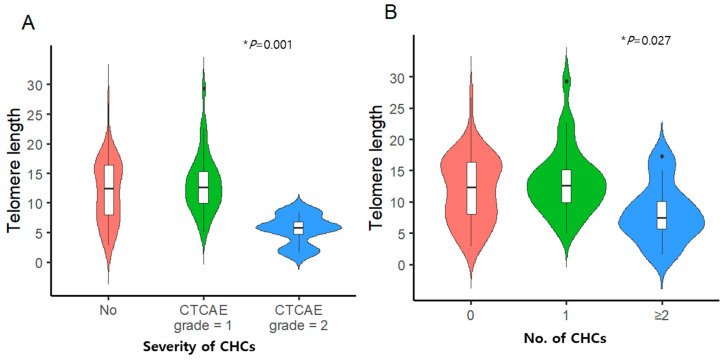
Telomere length according to the (**A**) severity of and (**B**) number of chronic health conditions (CHCs), * *p* < 0.05.

CHCs were diagnosed in 40 patients, including 17 with dyslipidemia, 10 with hormone deficiencies, 9 with ophthalmologic complications, 8 with obesity (defined as a body mass index (BMI) of ≥30), 5 with diabetes, and 1 with a secondary malignancy. Thirty-two (36.4%) and eight (9.1%) patients were classified as having grade I and II CHCs, respectively. None of the patients had grade III CHCs. No CHC influenced the RTL; however, patients with a grade II CHC had significantly shorter RTL than those with no or a grade I CHC (5.5% ± 2.5% vs. 11.9% ± 5.3% vs. 13.3% ± 5.1%, * *p* = 0.001) (Figure 5A). When the influence of multiple CHCs on RTL was examined, survivors with ≥2 CHCs had shorter RTL (* *p* = 0.027) (Figure 5B).

## 4. Discussion

Telomere length is widely considered a surrogate marker of aging, with shortened telomeres associated with higher risks of age-related illnesses that are often associated with chronic inflammation [11]. In this study, we performed an extensive analysis of the factors associated with RTL in 88 AYA survivors of childhood cancer. The study participants only included AYA survivors aged 15–35 years; therefore, the telomere lengths were compared in a relatively homogeneous group.

To begin with, the overall RTL in survivors at a median of 73 months post-therapy was not significantly shorter than the predicted value for age-matched references from a previous study [10], which is reassuring. No difference was found in the RTL between patients with solid tumors and those with hematologic malignancies. Data suggest that cytotoxic chemotherapy accelerates the aging process by inducing cellular senescence [12]. Previously, Song et al. [13] found that survivors of childhood cancers had significantly shorter telomeres compared to community controls. They included large numbers of 2427 survivors, mostly white and non-Hispanic, who were diagnosed between the ages of 0 and 23.6 years and had their DNA sampled between the ages of 6.0 and 66.4 years. In contrast, our study focused on a smaller cohort of 88 Korean AYA survivors diagnosed before the age of 19 years, with blood samples taken between the ages of 15 and 35 years. The difference in the findings might be partly due to the sample size, race, and ethnicity. However, our findings suggest that the impact of suffering childhood cancer on telomere might not be as significant in the AYA survivor population when compared to an age-matched reference; definitive conclusions have not yet been drawn. Several studies suggest that telomere shortening after cancer treatment may not be a permanent condition. There is a possibility of a recovery period during which the telomere length normalizes or even lengthens [6,14,15]. Another important factor to consider is that longer telomeres might be linked to a higher risk of childhood cancer [16,17]. It is probable that the childhood cancer patients in our study had a longer baseline telomere length compared to cancer-free individuals. Therefore, having a similar telomere length to cancer-free individuals later in life could indicate therapy-associated telomere attrition. Further examination of changes in telomere length in a larger cohort may provide more conclusive evidence.

An interesting aspect of our findings is the heterogeneity of RTL among different populations (Figure 1B). Some survivors exhibit telomere shortening, whereas others do not. The high degree of individual variation observed in this study suggests that the degree of remaining stem cell compensation for the loss of hematopoietic precursors may differ among individuals after treatment. Similar heterogeneity has been previously reported in a study of patients with breast cancer and pediatric cancers [18,19]. Cancer survivors who received chemotherapy, radiotherapy, and/or HSCT may experience premature aging due to various biological mechanisms. These include the formation of senescent cells, the shortening of telomeres, dysfunction in stem and progenitor cells, DNA damage, epigenetic alterations, and changes in microRNA expression [20]. Thus, the degree of aging may differ among patients.

No significant association was found between RTL and relapse in this study. However, in patients with hematological malignancies, relapse was associated with telomere shortening. Due to the small sample size, we could not determine whether relapse itself affected the risk of telomere attrition or whether HSCT influenced the telomere length.

Radiation has been widely recognized as a cause of acute cellular damage and can lead to telomere dysfunction [21]. Song et al. [13] found that chest and abdominal/pelvic radiotherapy was associated with shorter leukocyte telomere length in survivors, but not brain radiotherapy. This could be due to acute cellular damage from radiation therapy. However, it is also possible that there could be a possible association between specific treatment exposures and telomere length, partly influenced by biological differences in telomere profiles across different types of cancer [13]. Maeda et al. [22] found no overall statistically significant change in mean leukocyte telomere length associated with radiation therapy among 25 patients with solid tumors, and no difference was observed in the magnitude of telomere length change between cancer patients who received radiation therapy and a hospital-based comparison group. Baerlocher et al. [23] studied the impact of TBI on telomere length in 44 HSCT recipients and found no significant effect. In our study, we could not find any association between RTL and radiation therapy site or dose. There was also no correlation between telomere length and TBI in survivors who underwent allogeneic HSCT. Due to the limited sample size of our study, more research on a larger cohort is necessary to draw definite conclusions.

Several studies demonstrated telomere attrition shortly after HSCT, whose length eventually returned to normal [24,25]. Extensive telomere shortening in HSCT recipients is associated with higher all-cause mortality [26]. In our study, survivors who underwent allogeneic HSCT had a significantly shorter RTL than those who did not. The cause of the telomere shortening observed in our patients who underwent HSCT remains unclear. Telomere shortening after HSCT could be a consequence of the extensive cell proliferation required to achieve immune reconstitution. Another common hypothesis is that damage to normal cells caused by therapy can result in chronic systemic inflammation, leading to telomere attrition and premature aging [20]. In patients who have undergone HSCT, hematocytes have not been exposed to chemotherapy and, therefore, telomere shortening in these patients clearly reflects a process of accelerated aging. The impact of cellular DNA damage and senescence caused by cytotoxic drugs on the aging process of blood is still uncertain. It is possible that senescent cells could affect the surrounding microenvironment and non-senescent cells by secreting multiple cytokines, ultimately causing a small number of damaged cells to induce senescence [27,28]. If this hypothesis can be confirmed, identifying recipients at risk of cellular senescence could be a crucial aspect of the monitoring of long-term survivors after HSCT.

Patients who experienced grade II or higher acute GVHD had a significantly shorter RTL than those who did not. Similarly, patients with chronic GVHD had shorter RTL than those without GVHD, although this difference was not statistically significant. Baerlocher et al. [23] reported that chronic GVHD is associated with telomere attrition. Acute GVHD can cause an increased inflammation, which may accelerate the attrition of telomere length by promoting rapid cell turnover and/or by inducing the release of reactive oxygen species. This can result in direct damage to the telomeric DNA either via oxidative stress or the inhibition of telomerase activity [29]. Subsequent stabilization without telomere elongation is likely associated with the establishment of steady-state hematopoiesis. 

While short-term side effects from chemotherapy may be acceptable to achieve even a slight increase in survival rates, long-term toxicity, such as early-onset frailty or other signs of accelerated aging, can significantly impact one’s quality of life for several years. CHCs that develop after cancer therapy are common and frequently debilitating. Telomere reserves are potential biomarkers for various health-related conditions and can help regulate the biological processes associated with aging. In our study, the RTL was shorter in patients with severe or multiple CHCs. Chronic inflammation and oxidative stress are associated with telomere shortening [30]. In humans, this relationship has been demonstrated in patients with chronic inflammatory diseases, obesity, and diabetes mellitus. Our finding of telomere attrition in patients with severe or multiple CHCs is consistent with these previous studies. Similarly, Song et al. [13] reported an association between shorter telomere lengths and CHCs. Moreover, survivors with shorter telomere lengths were more likely to have multiple CHCs, as shown in our study; this association suggests that measuring the length of leukocyte telomeres, which is a biomarker for aging, could be a possible target for interventions to prevent CHCs in survivors of childhood cancer in future studies. Based on our findings, telomere length could serve as a valuable biomarker in the care of pediatric cancer survivors. It could help in the early detection of long-term health risks. The regular monitoring of telomere length could assist in managing the effects of chemotherapy and radiation, guiding personalized lifestyle and therapeutic interventions, and assessing bone marrow and stem cell health [31]. 

To prevent the shortening of telomeres in survivors, there are both pharmacological and non-pharmacological interventions that can be used. Pharmacological approaches include antioxidants such as vitamins C and E, anti-inflammatory drugs like aspirin, and hormone therapies like estrogen for women [32,33]. Non-pharmacological strategies include healthy lifestyle modifications such as diet, exercise, and sleep hygiene; stress reduction techniques like mindfulness and yoga; psychological support; nutritional supplements (e.g., omega-3 fatty acids and polyphenols); and minimizing exposure to environmental pollutants [30,34,35]. Previously, research has shown the association between unfavorable health behavior and shorter telomere length in survivors aged between 18 and 35 years, but not in older survivors [13]. Making healthy lifestyle choices and reducing exposure to environmental toxins could contribute to telomere maintenance and overall health by reducing oxidative stress and inflammation, which are key factors in telomere attrition [34,35,36]. Comprehensive research, clinical trials, and personalized approaches are essential to develop effective interventions. 

Our research on telomeres in pediatric cancer survivors shares some similarities and differences with previous studies [4,5,6,7,8,13]. These differences may be attributed to variations in study design, sample size, methods used to measure telomere length, age, and racial and socioeconomic background. There are some limitations in our study. First, this study included a relatively small number of patients who exhibited heterogeneity in terms of malignancy type, disease status, age, and type of treatment, which may have contributed to a lack of statistical power. A prospective study with a larger sample size should be conducted. It would also be interesting to measure terminal restriction fragment 2 (TRF2) expression using real-time qPCR, as TRF2 prevents telomere shortening [1,37]. Second, our study did not include a control group. It was practically difficult to conduct blood sampling in healthy AYAs. Instead, we compared the data of our participants to age-matched references from a study by Behrens et al. [10], which employed the same methods to measure RTL as in this study. Third, this was not a longitudinal study; therefore, we were unable to identify the changes in the RTL in individual patients. To draw solid conclusions about the impact of cancer treatment on the length of hematopoietic cell telomeres, it is necessary to measure the individual telomere lengths in long-term survivors and correlate the results with chromosomal instability data. Finally, we did not evaluate the telomere length of donors in cases where allogeneic HSCT was performed. It is possible that the telomere length of the donor could be reflected in the recipient’s leukocytes. Typically, childhood cancer patients receive HSCT from an older donor, which could result in shortened telomere length. However, our study found no significant difference in the age distribution between the donors and patients upon study enrollment, suggesting that age-related differences in telomere length may not be a significant concern. To draw definitive conclusions, it may be beneficial to conduct research using PB samples from transplanted patients and their respective donors, as well as buccal cells from transplanted patients obtained simultaneously, as the leukocytes of patients will primarily be derived from donor cells and reflect the donor’s RTL.

## 5. Conclusions

With the increasing number of pediatric cancer survivors, there is growing evidence that cancer treatments could cause premature aging, which may heighten the probability of non-cancer aging-related illnesses and adversely affect the quality of life for cancer survivors. The RTL in the overall pediatric cancer survivor population was not significantly shorter than the predicted values for age-matched reference. However, HSCT recipients showed RTL attrition compared to those who did not receive HSCT, and GVHD augmented RTL attrition. The RTL was shorter in patients with severe or multiple CHCs. Further research on the mechanisms that cause varying rates of telomere attrition may contribute to the development of possible approaches to lessen or even prevent this phenomenon. Although there is currently no consensus on the extent and clinical significance of the accelerated shortening of the RTL, the RTL may be a potential biomarker for determining severe or multiple CHCs in pediatric cancer survivors. Patients with an RTL below a certain threshold (yet to be accurately determined) may require special consideration during long-term follow-up care.

## Figures and Tables

**Table 1:** Demographic data and characteristics of the participants (*n* = 88).

Characteristics	No. (%) or Median (Range)
Sex	
Male	47 (53.4)
Female	41 (46.6)
Diagnosis	
Hematologic malignancy	
Acute lymphoblastic leukemia	21 (23.9)
Myeloid leukemia, myelodysplastic syndrome	10 (11.4)
Lymphoma	12 (13.6)
Solid tumor	
Brain tumor	16 (18.2)
Sarcoma	14 (15.9)
Germ cell tumor	12 (13.6)
Others	3 (3.4)
Age at diagnosis, y	13.0 (1–19)
Age at blood sampling, y	22.5 (15–34)
BMI at blood sampling, kg/m^2^	22.3 (14.8–35.3)
Relapse	
Yes	14 (15.9)
No	74 (84.1)
Duration off cancer therapy, mo.	73 (12–184)
Chemotherapy	88 (100)
Radiotherapy	38 (43.2)
Transplantation	
Allogeneic	15 (17.0)
Autologous	6 (6.8)
Chronic health conditions	
Dyslipidemia	17 (19.3)
Hormone deficiency	10 (11.4)
Ophthalmologic complications	9 (10.2)
Obesity, BMI ≥ 30	8 (9.09)
Diabetes	5 (5.7)
Secondary cancer	1 (1.1)

BMI, body mass index.

## Data Availability

The data presented in this study are available upon request from the corresponding author. The data are not publicly available due to privacy and ethical restrictions.
